# Association of high intra-patient variability in tacrolimus exposure with calcineurin inhibitor nephrotoxicity in kidney transplantation

**DOI:** 10.1038/s41598-023-43755-x

**Published:** 2023-10-02

**Authors:** Hyokee Kim, Ahram Han, Sanghyun Ahn, Seung-Kee Min, Jongwon Ha, Sangil Min

**Affiliations:** 1 Department of Surgery, Korea University College of Medicine, Seoul, South Korea; 2 Department of Surgery, Seoul National University College of Medicine, Seoul, South Korea; 3 Division of Transplantation and Vascular Surgery, Department of Surgery, Seoul National University Hospital, 101, Daehak-ro, Jongno-gu, Seoul, 03080 South Korea

**Keywords:** Immunology, Medical research

## Abstract

Tacrolimus intra-patient variability (IPV) is a novel predictive marker for long-term kidney transplantation outcomes. We examined the association between IPV and calcineurin inhibitor (CNI) nephrotoxicity and the impact of pharmacogenes on CNI nephrotoxicity and IPV. Among kidney transplant recipients at our hospital between January 2013 and December 2015, the records of 80 patients who underwent 1-year protocol renal allograft biopsy and agreed to donate blood samples for genetic analysis were retrospectively reviewed. The cohort was divided into the low and high IPV groups based on a coefficient variability cutoff value (26.5%). In multivariate analysis, the IPV group was involved in determining CNI nephrotoxicity (HR 4.55; 95% CI 0.05–0.95; *p* = 0.043). The 5-year graft survival was superior in the low IPV group than in the high IPV group (100% vs 92.4% respectively, p = 0.044). Analysis of the time above therapeutic range (TATR) showed higher CNI nephrotoxicity in the high IPV with high TATR group than in the low IPV with low TATR group (35.7% versus 6.7%, *p* = 0.003). Genetic analysis discovered that *CYP3A4* polymorphism (rs2837159) was associated with CNI nephrotoxicity (HR 28.23; 95% CI 2.2–355.9; p = 0.01). In conclusion, high IPV and CYP3A4 polymorphisms (rs2837159) are associated with CNI nephrotoxicity.

## Introduction

Since its introduction in the early 1990s, tacrolimus has become the backbone of immunosuppressive therapy in renal transplantation. The use of tacrolimus has been associated with better graft survival, a lower incidence of rejection, and fewer side effects compared to the use of cyclosporine^1^. However, tacrolimus has a narrow therapeutic range, with underexposure causing acute rejection and overexposure causing calcineurin inhibitor (CNI)-related toxicity^2,3^. In addition, a high level of inter-individual and intra-patient pharmacokinetic variability makes it difficult to control the concentration of tacrolimus^4^.

The intra-patient variability (IPV) of tacrolimus, which reflects the variability in tacrolimus exposure within individual patients, has been studied and has emerged as a novel clinical marker^5^. A high IPV is associated with inferior long-term outcomes of renal transplantation, including acute rejection, high chronicity scores, and graft loss^6–8^. However, studies demonstrating an association between tacrolimus IPV and CNI-related nephrotoxicity are lacking.

The pharmacokinetics of tacrolimus are widely known to be influenced by the *CYP3A5* phenotype. *CYP3A5* expressers require a tacrolimus dose that is 1.5–2 times higher than that of non-expressers to reach the same concentration in the blood. The Clinical Pharmacogenetics Implementation Consortium (CPIC) guidelines recommend that *CYP3A5* expressers receive a higher starting dose, whereas *CYP3A5* non-expressers should receive the standard starting dose^9^. Therefore, the various genetic polymorphisms in the genes encoding tacrolimus-metabolizing enzymes partly explain the inter-individual variability of tacrolimus. However, only a few studies have investigated the association between IPV and gene polymorphisms. A previous study confirmed that *CYP3A5* single-nucleotide polymorphisms (SNPs) did not affect IPV in pediatric kidney transplantation; however, IPV was associated with the T-cell-mediated rejection-free survival rate in the *CYP3A5* expresser group^10^. Moreover, Cheung et al. have reported that *CYP3A5* genotype and IPV were not related in Chinese patients who underwent kidney transplantation^11^. However, it is difficult to draw a clear conclusion because only a few studies have been published.

Therefore, this study aimed to evaluate the association between IPV and CNI nephrotoxicity and examine the effects of pharmacogenes on CNI nephrotoxicity and IPV.

## Methods

### Patients and immunosuppressive therapy

Among patients who received a kidney transplant at a university hospital between January 2013 and December 2015, 107 agreed to donate blood samples for genetic analysis. Among these, 80 patients who received a single kidney transplant, treated with a tacrolimus formulation (Prograf®, Astellas Pharma, Tokyo, Japan) twice daily, had three or more outpatient tacrolimus trough concentrations between 6 months and 1 year after transplantation, and underwent a protocol biopsy after 1 year were retrospectively reviewed. This study was conducted in accordance with the World Medical Association’s Declaration of Helsinki, and the protocol was approved by the institutional review board of the Seoul National University Hospital (IRB no. 1608-037-784). Written informed consent was obtained from all patients.

All patients received triple immunosuppression therapy consisting of tacrolimus, mycophenolate mofetil, and prednisolone. The target tacrolimus concentration range was 8–12 ng/mL in the 3-month post-transplantation period and 6–10 ng/mL between 3 months and 1 year after transplantation. Tacrolimus levels were measured monthly between 6 months and 1 year. A fixed dose of mycophenolate mofetil (500 mg) was administered twice daily. Intravenous prednisone 500 mg was administered during the operation, which was tapered off to a daily dose of 5 mg within the first month after transplantation. Basiliximab (20 mg 2 h before the operation and another 20 mg 4 days after) or anti-thymocyte globulin (1.5 mg/kg for 3 days; in high-risk patients, such as those with high PRA [> 80%] or positive donor-specific HLA antibody [DSA]) was used for induction therapy. To prevent the effect of CYP3A4/A5 inhibitor on drug metabolism, all patients were advised not to take grapefruit, anti-fungal agents, or calcium channel blockers.

Tacrolimus concentration was determined using a chemiluminescent microparticle immunoassay carried out using the Architect i2000SR immunoassay analyzer (Abbott, Chicago, IL, USA). The intraday coefficient of variation ranged from 2.4 to 3.5%. The accuracy was 96.0–100.0%. The interday coefficient of variability (CV) varied from 2.7 to 3.5%. The lower limit of quantification for tacrolimus was 0.8 ng/mL.

### Histological analysis

The hospital’s protocol biopsies were conducted 10 days and 1 year after kidney transplantation. We analyzed the data showing CNI nephrotoxicity identified in the 1-year protocol biopsy to confirm the effect of IPV and time in therapeutic range (TTR), which was calculated using the tacrolimus levels between 6 and 12 months.

The severity of histological lesions was recorded according to the Banff 2019 classification^12^. The CNI nephrotoxicity was assessed for both acute and chronic features. The histological features of acute CNI nephrotoxicity include early-stage hyalinization, dropout of individual myocytes in afferent arterioles, and isometric vacuolation of proximal straight tubules. We defined chronic CNI nephrotoxicity as biopsy findings showing the following: (1) high-grade arteriolar hyalinosis with luminal narrowing, (2) increasing glomerulosclerosis, and (3) tubulointerstitial damage^13–15^. The chronicity score was defined as the sum of ci, ct, cg, cv, ah, and mm^16^.

### Tacrolimus intra-patient variability

The tacrolimus concentration IPV was estimated by calculating the CV. The CV is a statistical measure of the degree of variation, which represents the ratio of the standard deviation (SD) to the mean tacrolimus concentration through the equation CV (%) = (SD/mean tac trough concentration) × 100^5^. Time weighted CV was also calculated using reported method. $TWu= \frac{1}{\mathrm{t}}{\Sigma }_{\mathrm{n}=1}^{\mathrm{i}}{\mathrm{x}}_{\mathrm{i}}{\mathrm{t}}_{\mathrm{i}}$ , TWσ = $\sqrt{\frac{1}{t}{\Sigma }_{\Pi =1}^{i}{\left({x}_{i}-\mu \right)}^{2}{t}_{i}}$, TWCV = $(TW\upsigma$  × 100)/TW $u$, where i is the patient’s visit to the outpatient clinic, x_i_ is the tacrolimus level (ng/mL) during the interval period, t_i_ is the time interval (days); defined as half the time interval between the measurement and the value preceding it plus half the time interval after the measurement, and t is the total duration of drug exposure (days)^17,18^.

Tacrolimus concentration was measured using whole blood drawn immediately prior to the morning dose of tacrolimus, and concentrations obtained from 6 months to 1 year after transplantation were analyzed. Data from the hospitalization periods were not considered. Erroneously high levels (> 20 ng/mL) of tacrolimus that were possibly measured soon after a dose was administered were excluded.

### Tacrolimus therapeutic range, time above and below therapeutic range

The Rosendaal linear interpolation method was used to calculate the TTR^19^. Using this method, we calculated the TTR with a tacrolimus therapeutic range of 6–10 ng/mL from 6 months to 1 year after transplantation. Additionally, the time above the therapeutic range (TATR) and the time below the therapeutic range (TBTR) were calculated using the linear interpolation method.

### Gene analysis

Using genomic DNA collected from a patient’s whole blood, targeted pharmacogene panel (ADME-PGx panel with 114 pharmacogenes) sequencing was conducted using the Illumina HiSeq 2500 platform^20^. Among 114 pharmacogenes, we identified 10 SNPs that have been reported to be associated with tacrolimus by PharmGKB^21,22^. The association between IPV and CNI nephrotoxicity in tacrolimus-related SNPs was further evaluated. We calculated the odds ratio (OR) for each SNP by analyzing the level of CNI nephrotoxicity or using logistic regression with penalized maximum likelihood.

### Statistical analysis

Statistical analysis was conducted using IBM SPSS Statistics for Windows, version 21.0 (IBM Corp., Armonk, NY, USA). All tests were two-tailed, and differences with *p* values of < 0.05 were considered statistically significant. Mean values were compared using Student’s t-test, and non-continuous variables were compared using the chi-squared test or Fisher’s exact test. To examine whether a high IPV was a risk factor for CNI nephrotoxicity, univariate and multivariate analyses using Cox regression were employed. RPART, a recursive partitioning procedure, or the tree classification algorithm in R 2.2.1 (R Development Core Team, Vienna, Austria) was used to identify the IPV cutoff point to analyze the relevance of IPV in CNI nephrotoxicity^23^. Based on this cutoff point, we classified the total cohort into the low and high IPV or TATR groups. One-way analysis of variance was used for comparisons between the groups according to IPV and TATR. The Kaplan–Meier curve was used to examine graft survival. Repeated ANOVA was used to compare between 10 days biopsy and 1 year biopsy results. Logistic regression analysis was performed for each gene to determine the extent to which each gene was associated with CNI nephrotoxicity or IPV. This gave test results for each gene rather than the results of multiple hypothesis tests; thus, no corrected *p*-value was presented. Collaboration with a clinical epidemiologist from Seoul National University was done to perform specialized statistical analysis at this point of the study.

## Results

### Entire cohort

Among 106 patients who provided informed consent, the following exclusion criteria was applied: death (n = 3), change in immunosuppression therapy (n = 3), or non-availability of 1-year protocol kidney biopsy results (n = 20). Consequently, the records of 80 patients were analyzed. The baseline characteristics of the entire study group are summarized in Table 1. The mean ages of kidney recipients and donors were 46.2 ± 14.0 and 48.0 ± 12.6 years, respectively. Thirty-five (43.8%) patients received kidneys from deceased donors, and 12 cases (15.0%) had ABO incompatibility. Three patients (3.8%) received a previous kidney transplant. Basiliximab was mostly used (95%) for induction therapy.

**Table 1:** Baseline characteristics of the entire group.

Characteristics	Entire group (n = 80)	Low IPV group (n = 53)	High IPV group (n = 27)	*p*-value
Recipient				
Age (years)	46.2 ± 14.0	44.8 ± 14.2	48.9 ± 13.4	0.217
Sex (male), %	46 (57.5)	31 (58.5)	15 (55.6)	0.802
Cause of ESRD (%)				0.394
Hypertension	5 (6.3)	4 (7.5)	1 (3.7)	
Diabetes mellitus	20 (25.0)	15 (28.3)	5 (18.5)	
Glomerulonephritis	31 (38.8)	22 (41.5)	9 (33.3)	
Cystic kidney disease	4 (5.0)	2 (3.8)	2 (7.4)	
Others	8 (10.0)	5 (9.4)	3 (11.1)	
Unknown	12 (15.0)	5 (9.4)	7 (25.9)	
Donor				
Age (years)	48.0 ± 12.6	47.4 ± 13.0	49.3 ± 11.8	0.526
Sex (male), %	38 (47.5)	26 (49.1)	12 (44.4)	0.981
No. of HLA mismatches	4.0 ± 1.8	3.7 ± 1.74	4.5 ± 1.7	0.046
Deceased donor (%)	35 (43.8)	21 (39.6)	14 (51.9)	0.297
Retransplantation (%)	3 (3.8)	3 (5.7)	0 (0)	0.208
ABO incompatible (%)	12 (15.0%)	8 (15.1)	4 (14.8)	0.974
Induction therapy (%)				
None	3 (3.8%)	3 (5.7)	0 (0)	0.363
Basiliximab	76(95.0)	49 (92.5)	27 (100)	
ATG	1 (1.3)	1 (1.9)	0 (0)	
CV (%)	22.7 ± 12.3	16.2 ± 5.0	35.5 ± 12.4	< 0.001
Time-weighted CV (%)	20.4 ± 11.4	14.7 ± 4.5	31.5 ± 12.7	< 0.001
Mean tacrolimus concentration	7.1 ± 1.11	7.0 ± 1.2	7.2 ± 1.0	0.44
between 6 months and 1 year (ng/mL)				
TTR (%)	68.1 ± 24.5	72.5 ± 26.2	59.5 ± 18.5	0.023
TATR (%)	5.1 ± 9.0	3.5 ± 8.7	8.1 ± 8.8	0.028
TBTR (%)	26.8 ± 24.5	24.0 ± 27.5	32.4 ± 19.2	0.159
Number of tacrolimus concentration measurements	6.4 ± 1.7	6.1 ± 1.6	6.8 ± 1.9	0.081

Data are expressed as means ± standard deviations or frequencies (percentages).

*TTR* time in therapeutic range, *TATR* time above therapeutic range, *TBTR* time below therapeutic range, *CV* coefficient of variability, *DSA* donor-specific alloantibody.

The frequency of tacrolimus concentration measurements in the entire cohort was 6.4 ± 1.7. The mean tacrolimus concentration CV and time-weighted CV in the 80 patients were 22.7 ± 12.3% and 20.4 ± 11.4%, respectively. The mean tacrolimus trough concentration between 6 and 12 months after transplantation was 7.1 ± 1.1 ng/mL. In that period, the mean TTR, TATR, and TBTR were 68.1 ± 24.5%, 5.1 ± 9.0%, and 26.8 ± 24.5%, respectively (Table 1).

We found 11 cases (13.8%) of histological CNI toxicity at the 1-year protocol biopsy in the entire cohort. All cases were related to the acute, and not chronic, features of CNI toxicity.

### Pharmacogenes associated with calcineurin inhibitor toxicity or intra-patient variability of tacrolimus exposure

In each patient, 114 pharmacogenes and 3,185 variants were identified. Among these, variants reported to be related to tacrolimus pharmacokinetics in PharmGKB were evaluated. We obtained the OR of the gene, with CNI nephrotoxicity as a dependent variable. *CYP3A4* (rs2837159) (HR 25.5; 95% CI 2.4–275.2; *p* = 0.008) polymorphisms showed an association with CNI nephrotoxicity (Table 2).

**Table 2:** Single-nucleotide polymorphism associated with calcineurin inhibitor toxicity in multiple analyses.

rs number	Gene name	Hazard ratio	95% CI	*p*-value
rs1045642	*ABCB1*	3.61	0.17–77.32	0.411
rs1128503	*ABCB1*	0.98	0.27–3.51	0.974
rs15524	*CYP3A5*	2.11	0.59–7.63	0.254
rs2032582	*ABCB1*	7.67	0.40–148.55	0.178
rs2276707	*NR1I2*	0.67	0.16–2.53	0.550
rs28371759	*CYP3A4*	25.50	2.36–275.18	0.008
rs3740066	*ABCC2*	3.08	0.82–11.56	0.096
rs776746	*CYP3A5*	1.67	0.46–6.04	0.437
rs890293	*CYP2J2*	3.61	0.58–22.62	0.170

*SNP* single-nucleotide polymorphism, *CNI* calcineurin inhibitor, *CI* confidence interval.

### Multivariate analysis

A CV cutoff point of 26.5% was determined to identify its association with CNI nephrotoxicity at 1-year protocol biopsy by employing RPART. To confirm the statistical significance of this result, we performed univariate and multivariate analyses of CNI nephrotoxicity. In the univariate and multivariate analyses, the high IPV group and CYP3A4 (rs2837159) were the risk factors associated with CNI nephrotoxicity (high IPV; HR 4.55; 95% CI 0.05–0.95; p = 0.043) (CYP3A4; HR 28.23; 95% CI 2.24–355.9; p = 0.01) (Table 3).

**Table 3:** Univariate and multivariate analysis of CNI nephrotoxicity.

Characteristics	Univariate			Multivariate		
	Odd ratio	(95% CI)	p value	Odd ratio	(95% CI)	p value
Recipient age (years)	1.01	(− 0.04 to 0.05)	0.76			
Donor age (years)	1.01	(− 0.04 to 0.06)	0.648			
DDKT	2.56	(− 0.38 to 2.26)	0.162			
ABO incompatible (%)	0.53	(− 2.79 to 1.51)	0.56			
CV (%), continuous	1.02	(− 0.02 to 0.07)	0.345			
CV						
Low IPV	Ref					
High IPV	4.29	(0.12 to 2.79)	0.032	4.55	(0.05–0.95)	0.043
Time-weighted CV (%), continuous	1.02	(− 0.03 to 0.07)	0.430			
Mean tacrolimus concentration between 6 months and 1 year (ng/mL)	0.98	(− 0.64 to 0.59)	0.938			
TTR (%)	0.99	(− 0.04 to 0.02)	0.591			
TATR (%)	1.01	(0.95 to 1.1)	0.752			
TBTR (%)	1.01	(0.98 to 1.03)	0.516			
CYP3A4 rsrs2837159	25.50	(2.36 to 75.19)	0.008	28.23	(2.24–355.9)	0.01

*TTR* time in therapeutic range, *TATR* time above therapeutic range, *TBTR* time below the therapeutic range, *CNI* calcineurin inhibitor, *CI* confidence interval, *CV* coefficient of variability, *CV* (low intra-patient variability [IPV] vs high IPV) cutoff value = 26.47.

### Low versus high intra-patient variability group

On a statistical basis with multivariate analysis, we divided the entire cohort into the low and high IPV groups using 26.5% of the cohort as the cutoff point for the CV. The low and high IPV groups consisted of 53 (66.3%) and 27 (33.7%) patients, respectively. No differences were found between the two groups, except for the CV, time weighted CV, and number of HLA mismatches (Table 1). The CV was 16.2 ± 5.0% and 35.5 ± 12.4% in the low and high IPV groups, respectively (*p* < 0.001). The time-weighted CV was 14.7 ± 4.5% and 31.5 ± 12.7% in the low and high IPV groups, respectively (*p* < 0.001). The high IPV group had a larger number of HLA mismatches than did the low IPV group (4.5 ± 1.7 vs 3.7 ± 1.7, *p* = 0.046). In the low IPV group, more patients were included the in time in therapeutic range (TTR) than was the case in the high IPV group (59.5 ± 18.5%vs. 72.5 ± 26.2%, *p* = 0.023). The proportion of TATR was higher in the high IPV group than in the low IPV group (8.1 ± 8.8% vs. 3.5 ± 8.7%, *p* = 0.028). No differences were observed in the distribution of polymorphisms between the high and low IPV groups (Supplementary Table 1).

### Clinical outcomes

We evaluated acute rejection and CNI nephrotoxicity in both groups. Acute rejection at the 10-day biopsy showed no difference between the two groups (7.5% versus 7.4%, *p* = 0.982). Nine patients had indication biopsy between transplantation and 1-year protocol biopsy. In four of them, borderline acute t-cell-mediated rejection occurred. Two of them were in the low IPV group, and the remaining were in the high IPV group. Two patients had acute T-cell-mediated rejection, and all of them were in the low IPV group. Only one patient had acute CNI nephrotoxicity in indication biopsy.

**Figure 1:**
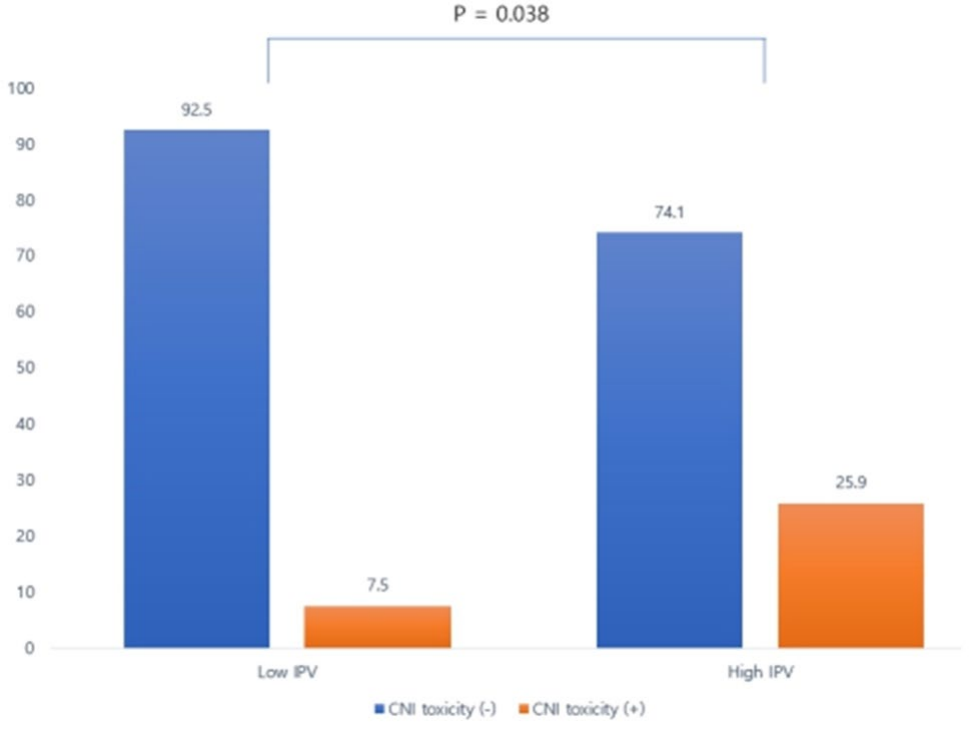
Different ratios of calcineurin inhibitor nephrotoxicity between the low and high intra-patient variability groups. *CNI* calcineurin inhibitor, *IPV* intra-patient variability.

Acute rejection at the 1-year biopsy showed no difference between the two groups (5.7% versus 7.4%, *p* = 0.760). However, at the 1-year protocol biopsy, the high IPV group had more acute CNI nephrotoxicity than did the low IPV group (25.9% vs 7.5%, *p* = 0.038) (Fig. 1). Glomerulosclerosis increased from 4.2 ± 4.7 to 9.2 ± 8.3% at 1 year in high IPV and remained stable from 4.6 ± 7.5 to 4.3 ± 6.2%. The delta value was significantly different (delta Glomerulosclerosis; 5.0 ± 7.8 vs − 0.4 ± 6.3; p = 0.001). Tubulointerstitial damage increased in both groups from 2 weeks to 1 year, with no significant difference (p = 0.072). No difference was found in histologic findings of hyalinosis from 2 weeks to 1 year and delta hyalinosis score (p = 0.478; p = 0.412). The delta chronicity score was significantly different between two groups, which means chronicity score was higher in the high IPV group than in the low IPV group (1.2 ± 1.5 vs 0.5 ± 1.4; p = 0.046).

For long-term outcome, development of de novo DSA was not different between IPV groups (p = 0.536). Among 80 patients, 2 patients had de novo DSA. One of them was in the low IPV group and the other was in the high IPV group. The 5-year graft survival in the low IPV group was superior compared with the high IPV group (100% vs 92.4% respectively, p = 0.044) (Fig. 3A). When group was categorized by CNI toxicity reported in 1-year biopsy, the 5-year graft survival was also not different between the two groups (p = 0.568).

### Intra-patient variability and time above therapeutic range

**Figure 2:**
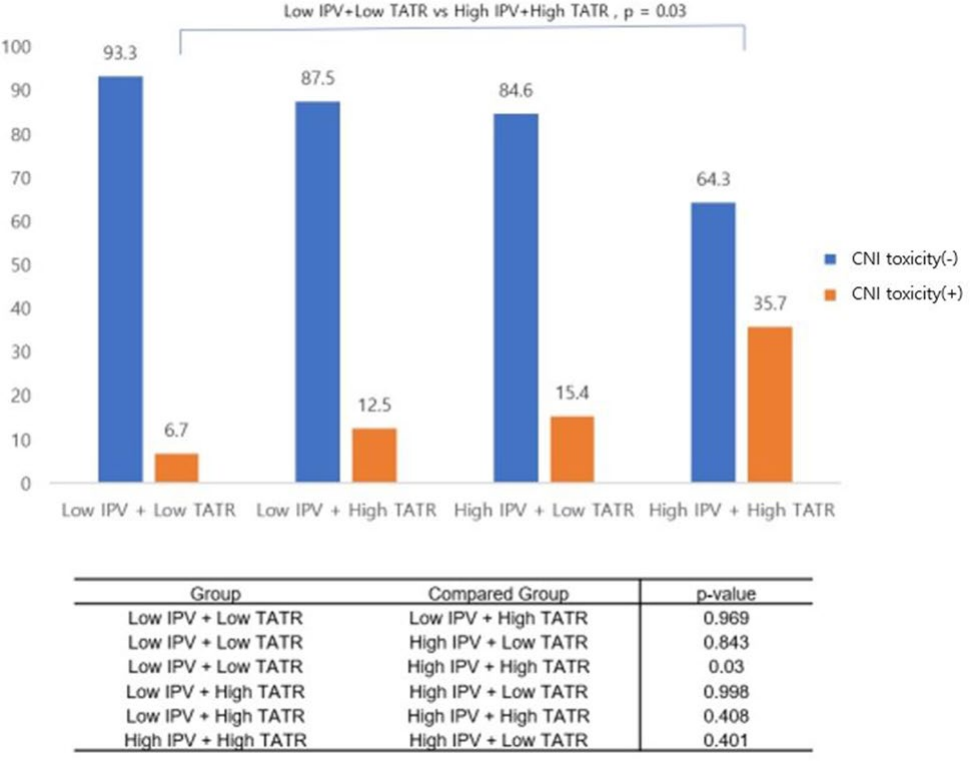
Different ratios of calcineurin inhibitor toxicity when divided by time above therapeutic range and intra-patient variability. *CNI* calcineurin inhibitor, *IPV* intra-patient variability, *TATR* time above therapeutic range.

**Figure 3:**
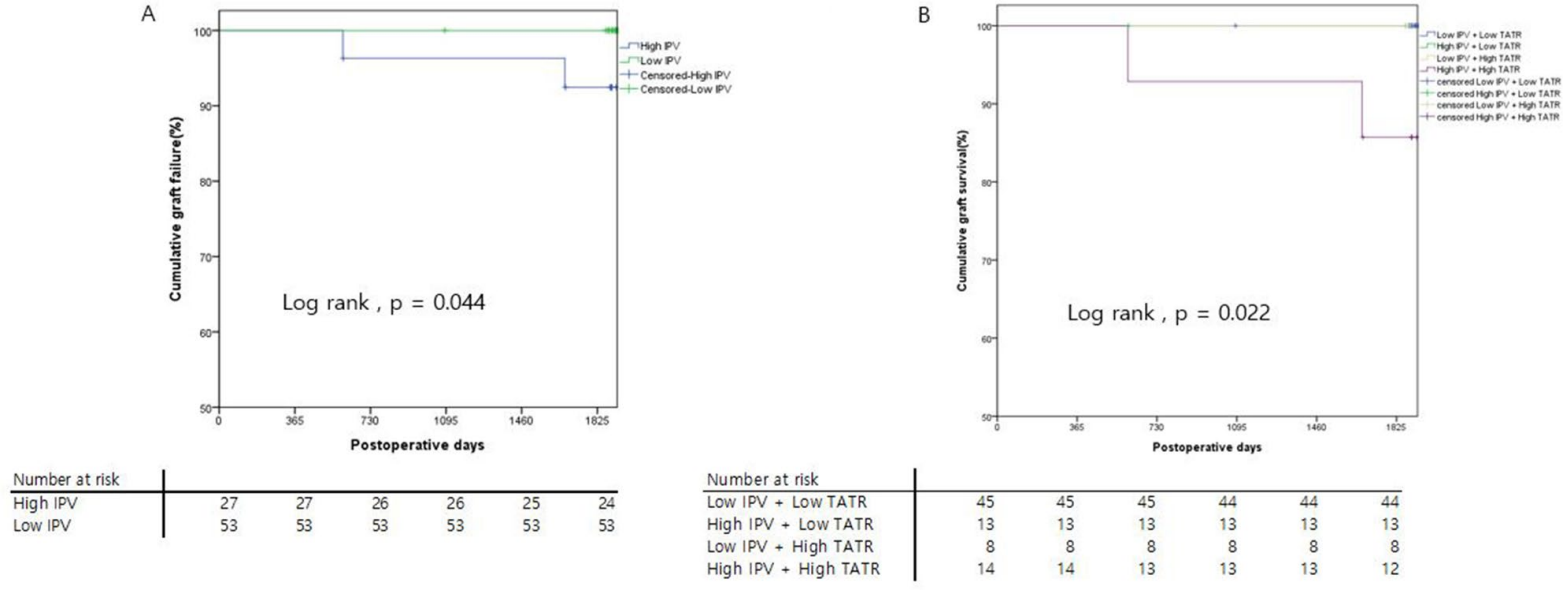
(**A**) Graft survival when divided by intra-patient variability. (**B**) Graft survival when divided by time above therapeutic range and intra-patient variability. *IPV* intra-patient variability, *TATR* time above therapeutic range.

We used the RPART method to calculate the cutoff TATR value and analyze its association with CNI toxicity. A TATR of 6.55% was chosen as the cutoff point. Then, we classified the entire cohort into four groups based on TATR and IPV. High IPV + high TATR (n = 14), high IPV + low TATR (n = 13), low IPV + low TATR (n = 45), and low IPV + high TATR (n = 8) groups showed 35.7%, 15.4%, 6.7%, and 12.5% CNI toxicity, respectively. There was more CNI nephrotoxicity in the high IPV with high TATR group than in the low IPV with low TATR group (35.7% vs 6.7%, *p* = 0.003) (Fig. 2). The 5-year survival of the high IPV with high TATR group was 86%, which was statistically inferior to that of other groups which showed 100% (p = 0.022) (Fig. 3B).

## Discussion

Since Borra et al. have reported that high IPV in tacrolimus exposure has a negative effect on the long-term outcomes of kidney transplantation, other studies have reported an association between high IPV and poor clinical outcomes^24–26^. Similarly, we showed that high IPV was associated with poor long-term outcomes and early deterioration of chronic histological lesions in adult and pediatric patients^6,7,10^. However, there has been little evidence to support an association between IPV and CNI nephrotoxicity.

Using a CV cutoff value of 26.5%, our findings revealed that there was more CNI nephrotoxicity in the high IPV group than in the low IPV group at the 1-year protocol biopsy. In addition, IPV was associated with CNI nephrotoxicity by multivariate analysis. Similar to our results, Turgut et al. have shown that high IPV was related to BK virus-associated nephropathy and chronic CNI toxicity, which were considered overimmunosuppression states^27^. However, it is difficult to directly state that high IPV alone is an overimmunosuppression state. Park et al. have revealed that IPV and tacrolimus C0 levels in the 1st year post transplantation were related to CNI toxicity^17^. In the present study, this was further explained using IPV and TTR.

We showed that patients with high IPV and high TATR had significantly higher CNI nephrotoxicity than those with low IPV and low TATR. We assumed that TTR was one of the reasons for the high level of CNI toxicity in the high IPV group. TTR has recently been studied as an indicator of how appropriate a patient’s drug concentration is in relation to the target tacrolimus range^28,29^. There was more TATR among patients in the high IPV group than among those in the low IPV group. Moreover, the low IPV group had a higher TTR rate than did the high IPV group. According to subgroup analysis, the high IPV and high TATR groups had higher CNI toxicity than did the low IPV and low TATR groups. However, the low IPV and high TATR groups did not show a higher ratio of CNI toxicity than did the other groups. Although the difference in the number for each group was a limitation of this study, our results suggest that the interaction between high IPV and high TATR may be associated with CNI nephrotoxicity.

A question of whether the CV cutoff point of 26.5% was appropriate may be raised. To analyze the relationship between IPV and clinical outcomes, other studies have also divided cohorts into low and high IPV groups. However, no specific criteria were used. Some studies have used the median CV value to divide a cohort into these two groups^6,7,24,30^. Vanhove et al. have divided their cohort into three equal parts^31^. The cutoff values in other studies differed, ranging from 14.4 to 20.5%^6,7,24,31^. We used RPART incorporating logistic regression to generate a statistical basis for the selection of a CV cutoff point of 26.47%; however, this value was higher than those employed in other studies. To verify our results, whether the same result is obtained from the same CV value in a large population study should be determined.

Further, we investigated the level of acute rejection in the low and high IPV groups. Ro et al. have reported that more acute rejection occurred in the high IPV group than in the low IPV group^6^. However, in our study, there were no statistically significant differences between the two groups, which could be owing to the small sample size, short timeframe, and different CV cutoff point in our study. Our CV cutoff point was used to determine the difference in CNI nephrotoxicity. Therefore, the results based on the cutoff point may be different. Further studies are required to determine how the results of CNI toxicity and acute rejection may differ depending on which CV values are used.

There are several ways to obtain CVs. In addition to simply using the standard deviation to calculate the CV, recent papers have used dose-adjusted CV and time-adjusted CV^17,18^. In addition to the CV, we calculated the time-weighted CV, and the time-weighted CV was different between the high IPV group and the low IPV group. However, in the multivariate analysis, the time-weighted CV was not a significant variable affecting CNI toxicity. It remains unclear which CV is better to calculate; however, a recent paper has suggested that both time-weighted CV and CV reflect non-adherence^18^.

We found that *CYP3A4* polymorphism (rs2837159) affected the level of CNI nephrotoxicity. *CYP3A4* SNP, which is known to affect tacrolimus pharmacokinetics, is located in intron 10, with a transition from T to C at position 878. This mutation may increase the activity of the CYP3A4 enzyme, thereby increasing the tacrolimus clearance rate and decreasing the plasma drug concentration^32^. Increased *CYP3A4* activity can lead to high systemic or tissue concentrations of tacrolimus metabolites. Zegarska et al. have reported that tacrolimus metabolites may have nephrotoxic effects^33^. Therefore, our findings lead to the hypothesis that *CYP3A4* polymorphism increases the tacrolimus clearance rate and the concentration rate of tacrolimus metabolites, which in turn increases the risk of nephrotoxicity.

Our study did not show any association between CYP3A5 and CNI toxicity. In the CPIC guidelines published in 2015, it was recommended to increase the starting dose of tacrolimus by 1–2 times because the CYP3A5 expresser has a faster metabolism than does the non-expresser^9^. Although polymorphism in CYP3A5 showed an increase in the risk of acute rejection, few studies showed a link with CNI toxicity. We previously reported that tacrolimus pharmacokinetic profiles were very similar between CYP3A5 expressers and non-expressers after when target tacrolimus trough concentration was fully achieved^34^. Moreover, according to Gervasini et al., CYP3A4*1B has a significant impact on tacrolimus clearance in addition to the CYP3A5 genotype^35^. Our study included a small number of Korean recipients and compared CNI toxicity in 1-year biopsies; thus, it is believed to have different results from other papers. Studies with a larger population are warranted to confirm our findings.

Our study had some limitations. First, owing to its retrospective design, all subjects had different intervals at which their tacrolimus levels were measured. In addition, other factors known to be associated with IPV, such as nonadherence, food consumption, and drug–drug interactions, could not be analyzed owing to the absence of relevant records. Second, the entire cohort was a single ethnic group with a small sample size. For further statistical significance, a multivariate analysis with a large sample size would be required to identify risk factors. As other ethnic groups may have different pharmacogenes, studies using more varied samples are required. Additionally, our data have significant percentage of loss to follow-up and the small number of patients relate to the event rate. Further well-designed large number prospective study is warranted.

In conclusion, our data suggested that high IPV and *CYP3A4* polymorphism (rs2837159) may be associated with CNI nephrotoxicity.

### Supplementary Information

Supplementary Table 1.

## Data Availability

The datasets used and/or analyzed during the current study are available from the corresponding author on reasonable request.

## References

[1] Ekberg H (2007). Reduced exposure to calcineurin inhibitors in renal transplantation. N. Engl. J. Med..

[2] Mohammadpour N, Elyasi S, Vahdati N, Mohammadpour AH, Shamsara J (2011). A review on therapeutic drug monitoring of immunosuppressant drugs. Iran. J. Basic Med. Sci..

[3] Kershner RP, Fitzsimmons WE (1996). Relationship of FK506 whole blood concentrations and efficacy and toxicity after liver and kidney transplantation. Transplantation.

[4] Andrews LM (2017). Pharmacokinetic considerations related to therapeutic drug monitoring of tacrolimus in kidney transplant patients. Expert. Opin. Drug Metab. Toxicol..

[5] Shuker N, van Gelder T, Hesselink DA (2015). Intra-patient variability in tacrolimus exposure: Causes, consequences for clinical management. Transplant. Rev. (Orlando).

[6] Ro H (2012). Impact of tacrolimus intraindividual variability and CYP3A5 genetic polymorphism on acute rejection in kidney transplantation. Ther. Drug Monit..

[7] Mo H (2019). Association of intrapatient variability of tacrolimus concentration with early deterioration of chronic histologic lesions in kidney transplantation. Transplant. Direct.

[8] Whalen HR (2017). High intrapatient tacrolimus variability is associated with worse outcomes in renal transplantation using a low-dose tacrolimus immunosuppressive regime. Transplantation.

[9] Birdwell KA (2015). Clinical Pharmacogenetics Implementation Consortium (CPIC) guidelines for CYP3A5 genotype and tacrolimus dosing. Clin. Pharmacol. Ther..

[10] Choi JS (2022). Effects of tacrolimus intrapatient variability and CYP3A5 polymorphism on the outcomes of pediatric kidney transplantation. Pediatr. Transplant..

[11] Cheung CY (2019). Impact of CYP3A5 genetic polymorphism on intrapatient variability of tacrolimus exposure in Chinese kidney transplant recipients. Transplant. Proc..

[12] Loupy A (2020). The Banff 2019 kidney meeting report (I): Updates on and clarification of criteria for T cell- and antibody-mediated rejection. Am. J. Transplant..

[13] Liptak P, Ivanyi B (2006). Primer: Histopathology of calcineurin-inhibitor toxicity in renal allografts. Nat. Clin. Pract. Nephrol..

[14] Naesens M, Kuypers DR, Sarwal M (2009). Calcineurin inhibitor nephrotoxicity. Clin. J. Am. Soc. Nephrol..

[15] Nankivell BJ (2003). The natural history of chronic allograft nephropathy. N. Engl. J. Med..

[16] Mo H (2019). Association of intrapatient variability of tacrolimus concentration with early deterioration of chronic histologic lesions in kidney transplantation. Transplant. Direct..

[17] Park Y (2021). Intrapatient variability in tacrolimus trough levels over 2 years affects long-term allograft outcomes of kidney transplantation. Front. Immunol..

[18] Kostalova B (2022). Comparison of different methods to assess tacrolimus concentration intra-patient variability as potential marker of medication non-adherence. Front. Pharmacol..

[19] Rosendaal FR, Cannegieter SC, van der Meer FJ, Briët E (1993). A method to determine the optimal intensity of oral anticoagulant therapy. Thromb. Haemost..

[20] Yoon JG (2021). Unraveling the genomic architecture of the CYP3A locus and ADME genes for personalized tacrolimus dosing. Transplantation.

[21] Barbarino JM, Whirl-Carrillo M, Altman RB, Klein TE (2018). PharmGKB: A worldwide resource for pharmacogenomic information. Wiley Interdiscip. Rev. Syst. Biol. Med..

[22] Barbarino JM, Staatz CE, Venkataramanan R, Klein TE, Altman RB (2013). PharmGKB summary: Cyclosporine and tacrolimus pathways. Pharmacogenet. Genom..

[23] Faderl S (2005). Angiogenic factors may have a different prognostic role in adult acute lymphoblastic leukemia. Blood.

[24] Borra LC, Roodnat JI, Kal JA, Mathot RA, Weimar W, van Gelder T (2010). High within-patient variability in the clearance of tacrolimus is a risk factor for poor long-term outcome after kidney transplantation. Nephrol. Dial. Transplant..

[25] van Gelder T (2014). Within-patient variability in immunosuppressive drug exposure as a predictor for poor outcome after transplantation. Kidney Int..

[26] Sapir-Pichhadze R, Wang Y, Famure O, Li Y, Kim SJ (2014). Time-dependent variability in tacrolimus trough blood levels is a risk factor for late kidney transplant failure. Kidney Int..

[27] Turgut D (2021). Tacrolimus intrapatient variability in BK virus nephropathy and chronic calcineurin toxicity in kidney transplantation. Saudi J. Kidney Dis. Transplant..

[28] Song T (2019). Increasing time in therapeutic range of tacrolimus in the first year predicts better outcomes in living-donor kidney transplantation. Front. Immunol..

[29] Davis S (2018). Lower tacrolimus exposure and time in therapeutic range increase the risk of de novo donor-specific antibodies in the first year of kidney transplantation. Am. J. Transplant..

[30] Spierings N, Holt DW, MacPhee IA (2013). CYP3A5 genotype had no impact on intrapatient variability of tacrolimus clearance in renal transplant recipients. Ther. Drug Monit..

[31] Vanhove T, Vermeulen T, Annaert P, Lerut E, Kuypers DRJ (2016). High intrapatient variability of tacrolimus concentrations predicts accelerated progression of chronic histologic lesions in renal recipients. Am. J. Transplant..

[32] Li DY, Teng RC, Zhu HJ, Fang Y (2013). CYP3A4/5 polymorphisms affect the blood level of cyclosporine and tacrolimus in Chinese renal transplant recipients. Int. J. Clin. Pharmacol. Ther..

[33] Zegarska J (2016). Tacrolimus metabolite M-III may have nephrotoxic and myelotoxic effects and increase the incidence of infections in kidney transplant recipients. Transplant. Proc..

[34] Min SI (2010). CYP3A5 *1 allele: Impacts on early acute rejection and graft function in tacrolimus-based renal transplant recipients. Transplantation.

[35] Gervasini G (2012). Impact of genetic polymorphisms on tacrolimus pharmacokinetics and the clinical outcome of renal transplantation. Transplant. Int..

