# Supplementary material for: Association of high intra-patient variability in tacrolimus exposure with calcineurin inhibitor nephrotoxicity in kidney transplantation
Source: Sci Rep. 2023 Oct 2;13:16502. doi: 10.1038/s41598-023-43755-x (PMC10545770; doi:10.1038/s41598-023-43755-x)
Supplement: Supplementary file 1 — Supplementary Table 1. [file 41598_2023_43755_MOESM1_ESM.docx]

| Supplementary Table 1. SNPs associated with IPV in multiple SNP analyses | | | | |
| --- | --- | --- | --- | --- |
|  |  |  |  |  |
| rs number | Gene Name | Hazard Ratio | 95% CI | p value |
| rs1045642 | ABCB1 | 1.9 | 0.34-9.28 | 0.45 |
| rs1057868 | POR | 1.03 | 0.35-2.53 | 0.909 |
| rs1128503 | ABCB1 | 1.3 | 0.46-3.02 | 0.737 |
| rs15524 | CYP3A5 | 2.89 | 0.97-6.64 | 0.058 |
| rs2032582 | ABCB1 | 1.29 | 0.37-3.86 | 0.76 |
| rs2276707 | NR1I2 | 0.55 | 0.19-1.39 | 0.186 |
| rs28371759 | CYP3A4 | 2.04 | 0.29-16.31 | 0.453 |
| rs3740066 | ABCC2 | 1.32 | 0.56-3.76 | 0.437 |
| rs776746 | CYP3A5 | 2.35 | 0.77-5.36 | 0.15 |
| rs890293 | CYP2J2 | 4.44 | 0.42-11.83 | 0.351 |
|  |  |  |  |  |
| SNP. Single-nucleotide polymorphism; CNI, calcineurin inhibitor; CI, confidence interval; IPV, intra-patient variability | | | | |
